# Supplementary material for: Abrupt transitions in time series with uncertainties
Source: Nat Commun. 2018 Jan 3;9:48. doi: 10.1038/s41467-017-02456-6 (PMC5752700; doi:10.1038/s41467-017-02456-6)
Supplement: Supplementary file 1 — Supplementary Information [file 41467_2017_2456_MOESM1_ESM.pdf]

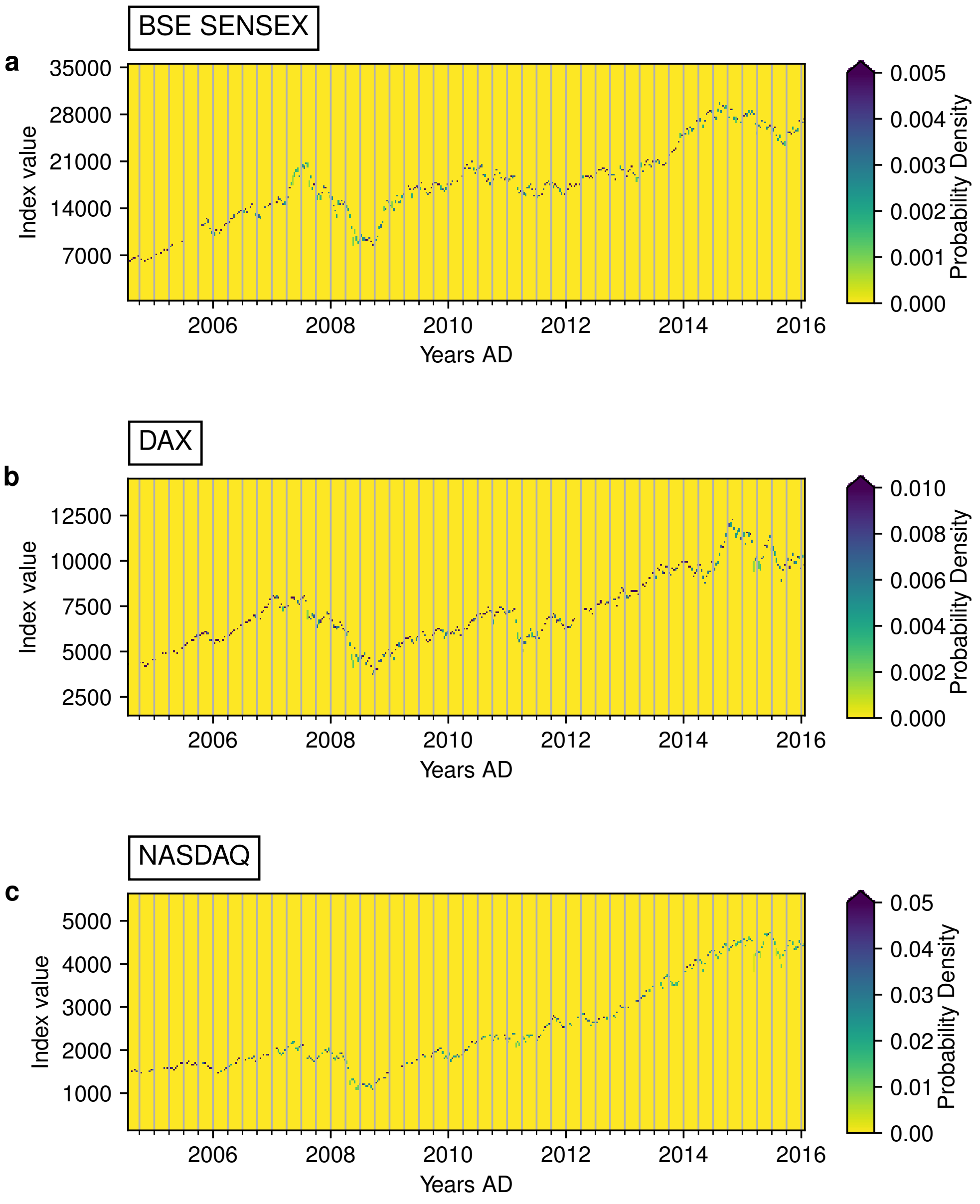

Supplementary Figure 1: Probability density time series: Stock indices. The probability density time series for the three stock indices shown as a colormap: (a) BSE SENSEX (Mumbai, India), (b) DAX (Frankfurt, Germany), and (c) NASDAQ (New York, USA). The densities are obtained by assuming a uniform random distribution of the index values bounded by the reported daily maximum and minimum values.

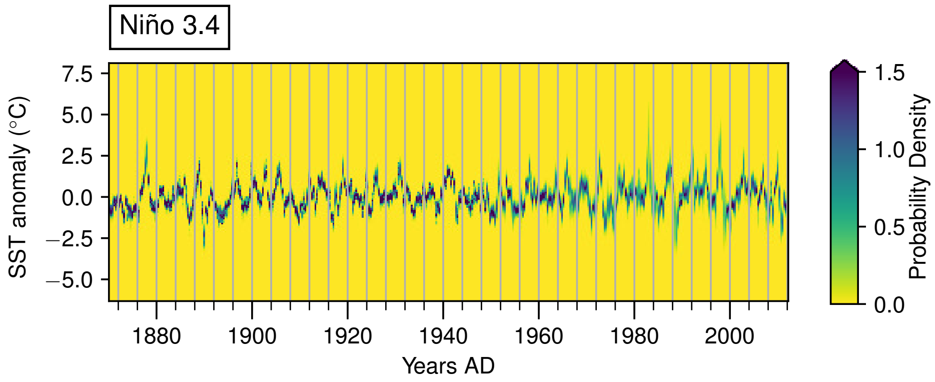

Supplementary Figure 2: Probability density time series: SST anomalies. The probability density time series obtained by applying kernel density estimates on the monthly SST values for all grid points in the Niño 3.4 region at each time point of observation.

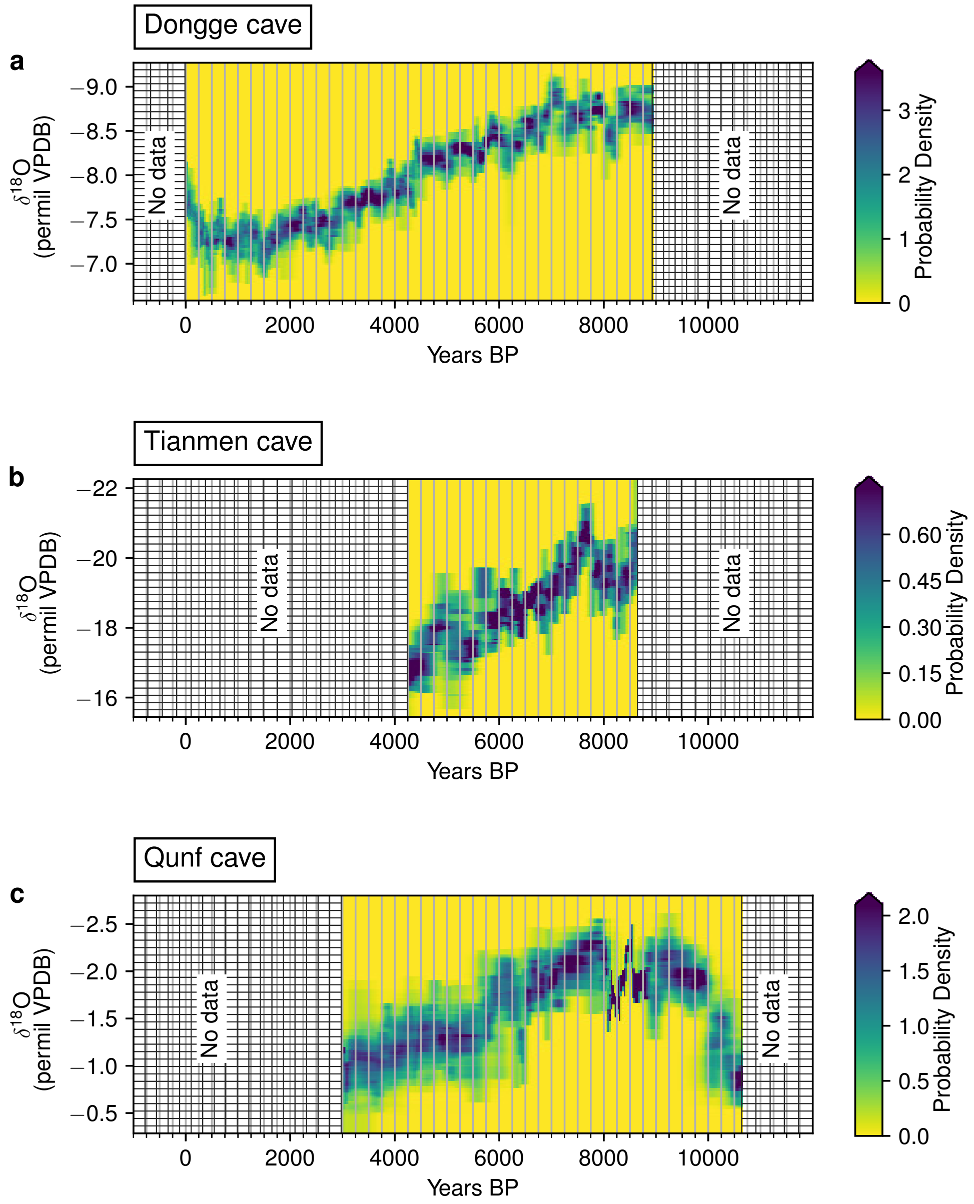

Supplementary Figure 3: Probability density time series: Paleoclimate proxies. The probability density time series estimated from the age–depth and proxy–depth measurements for the $\delta^{18}O$ isotope datasets obtained from three caves in the Asian monsoon domain: (a) Dongge cave in south China, (b) Tianmen cave in south China, and (c) Qunf cave in Oman. The uncertainty in these datasetes arise primarily from the imprecision in assigning ages to the $\delta^{18}O$ values measured along the depth of the stalagmites.

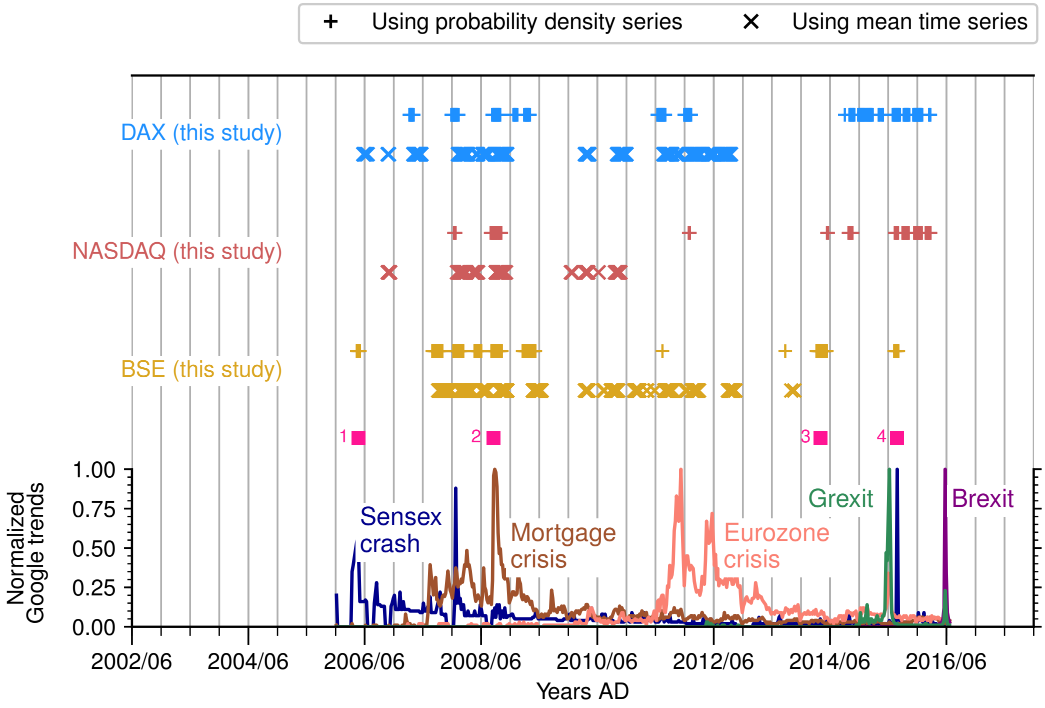

Supplementary Figure 4: Abrupt transitions in financial stock indices detected using the probability density time series (plus signs) and the mean time series (crosses). The bottom panel shows the normalized Google trends results for several relevant search terms. Additional pink squares correspond to: (1) BSE SENSEX crash of 22 May 2006, (2) bankruptcy claim by Lehman Brothers on 15 September 2009, and (3) Indian parliamentary elections from 7 April to 12 May 2014.

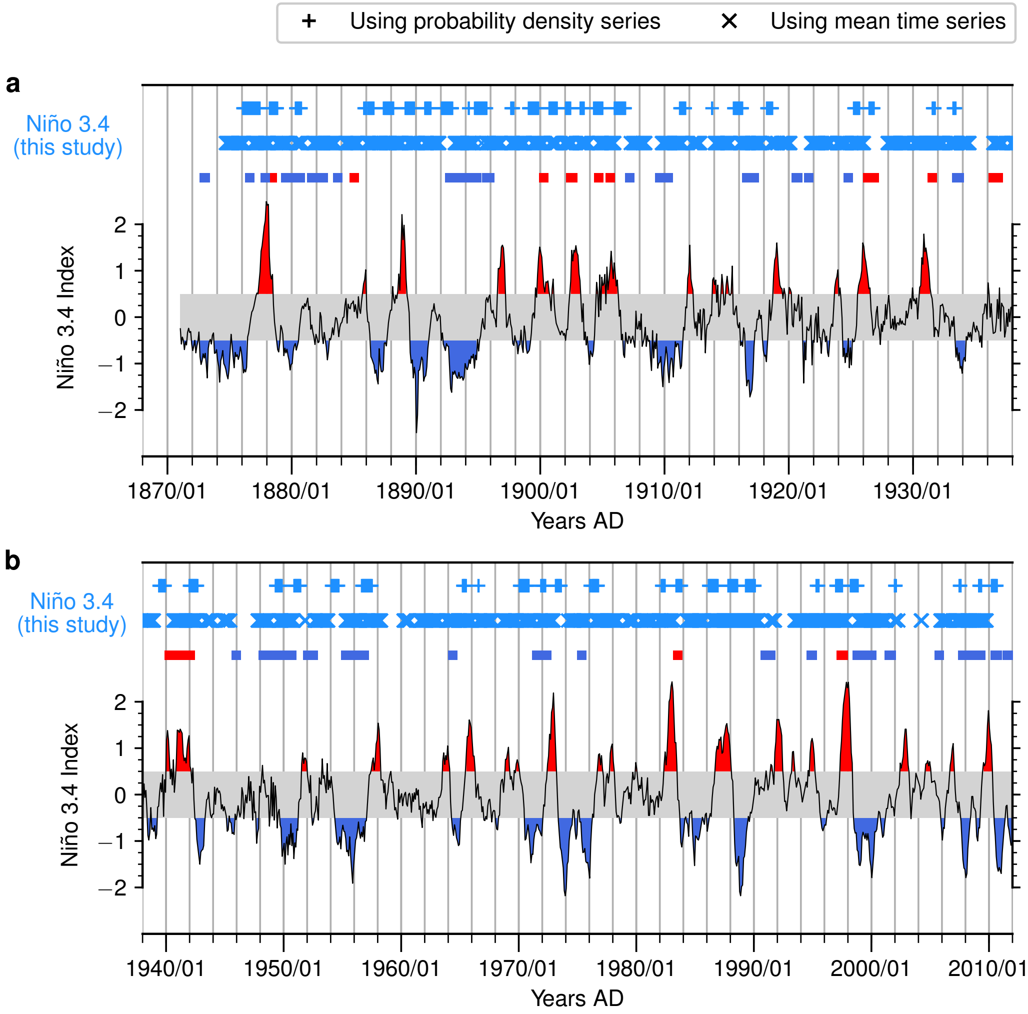

Supplementary Figure 5: Abrupt transitions in Niño 3.4 SST anomalies during the last 150 years, detected using the probability density time series (plus signs) and the mean time series (crosses): **(a)** The period from 1870 up to 1940, and **(b)** The period from around 1940 to 2012. Our results are compared to the Niño 3.4 index with red shaded regions denoting El Niño years and blue shaded regions denoting La Niña years. Extreme PDO states based on the NCEI PDO index are shown as little red (blue) squares corresponding to the positive (negative) phase of the PDO.

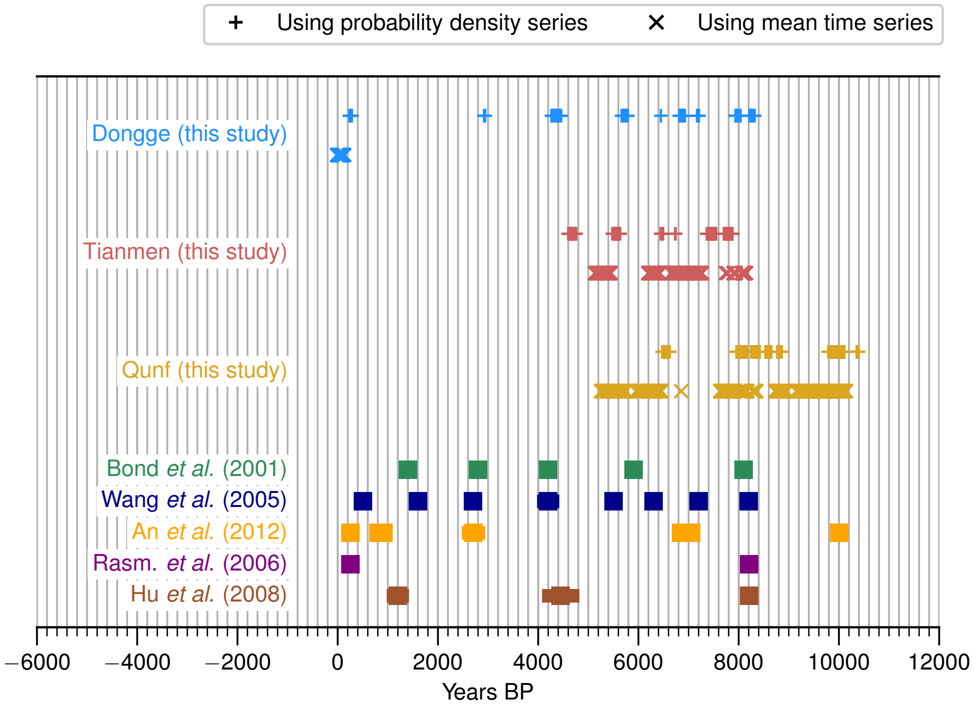

Supplementary Figure 6: Abrupt transitions in δ^{18}O isotopes detected using the probability density time series (plus signs) and the mean time series (crosses) for three caves in the Asian monsoon domain: (a) Dongge cave, (b) Tianmen cave, and (c) Qunf cave. The detected transitions are compared to previously reported events (squares) related to the weakening of the Asian monsoon during the Holocene.

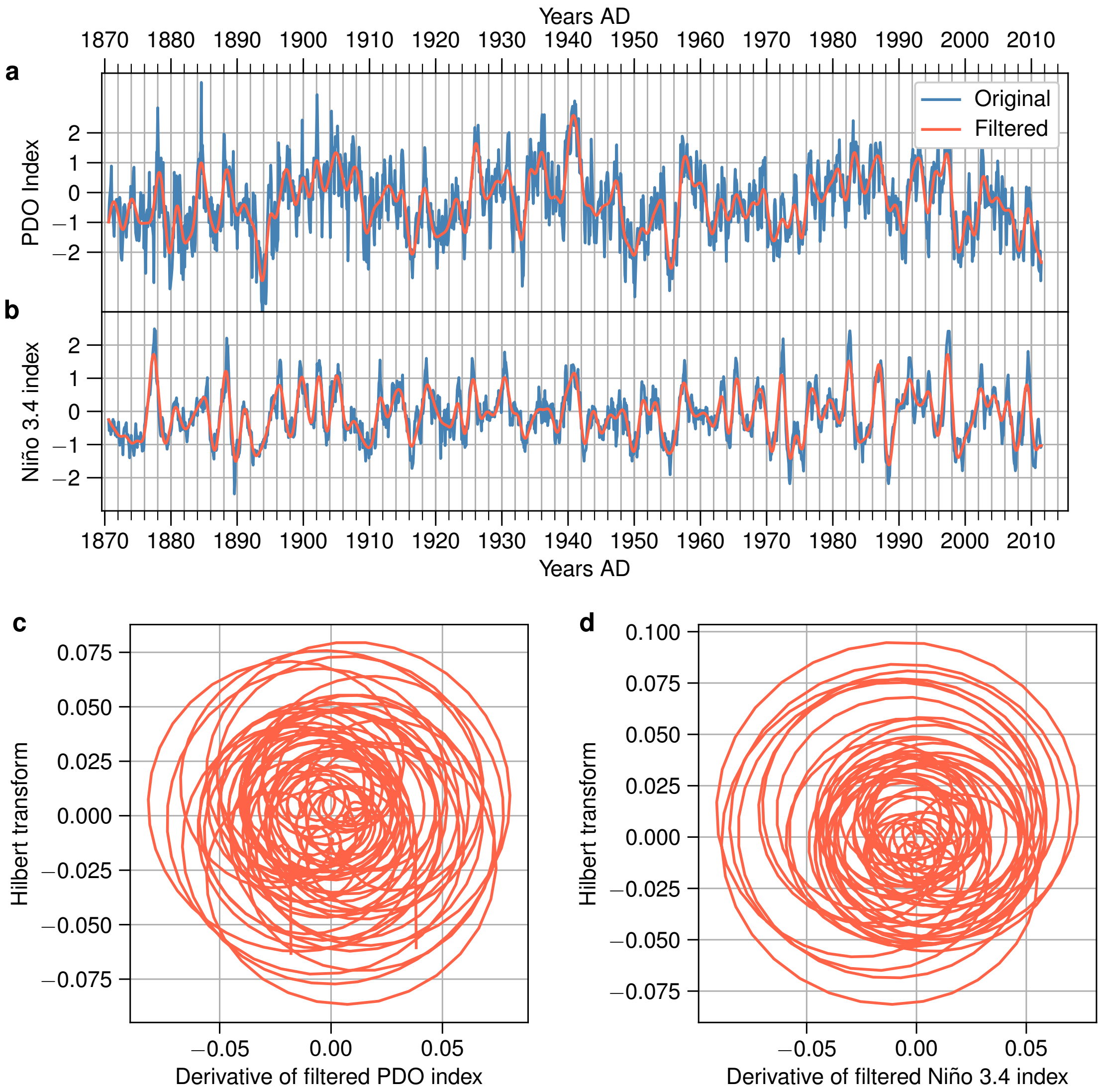

Supplementary Figure 7: Estimating the phase of PDO and Niño 3.4 indices using the filtered time series for each of the two indices (shown in **a** and **b**). The filtered time series were obtained using a low-pass forward-backward Butterworth filter that dampens all frequencies higher than $\frac{1}{12}$ month^{-1}. The filtered time series are then differentiated with respect to time to obtain the time derivative, which is used for the Hilbert transform (shown in **c** and **d**). The ratio of the Hilbert transform of the time derivative to the the time derivative is defined as the tangent of the instantaneous phase. The time derivative helps to ensure that the signal is phase coherent, i.e., it rotates (approximately) about a single center, as seen in **c** and **d**.

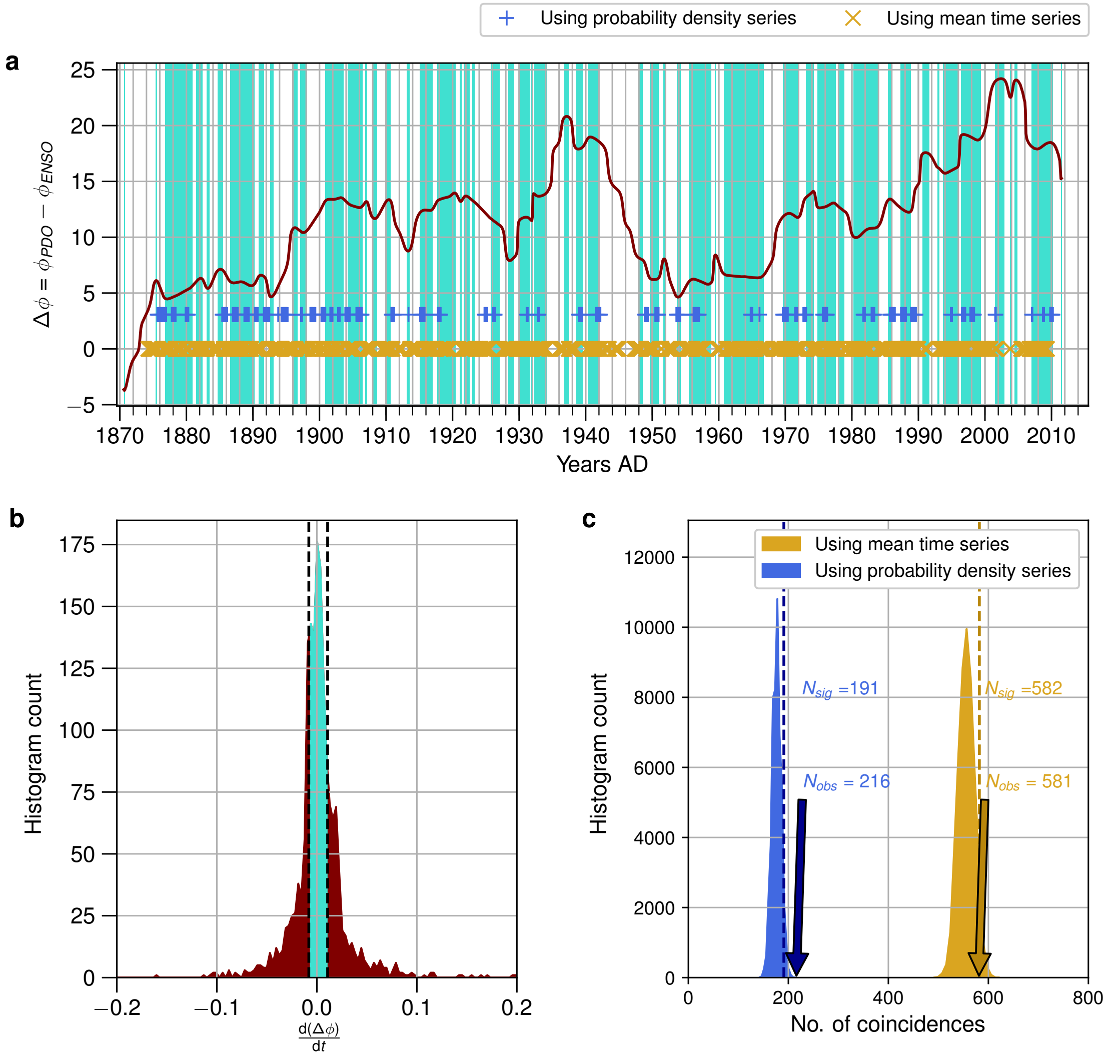

Supplementary Figure 8: Coincidence analysis between the detected transitions and the periods of phase locking between the PDO and the ENSO (shown as turquoise shaded regions in **a**) using the probability density time series (blue plus signs in **a**) and the mean time series (golden crosses in **a**). The periods of phase-locking are identified by first defining the phase difference between the individual phases $\phi_t PDO$ and ϕ_t^{ENSO} as $\Delta\phi_t = \phi_t^{PDO} - \phi_t^{ENSO}$. The phase-locking periods are defined as plateaus in $\Delta\phi_t$ where $\Delta\phi_t \approx 0$. In practice this is determined by identifying those time points where the time derivative of the difference $\frac{\mathrm{d}\Delta\phi_t}{\mathrm{d}t}$ fall between the 25-th and 75-th percentile of all obtained $\frac{\mathrm{d}\Delta\phi_t}{\mathrm{d}t}$ values (shown as a histogram in **b**). The interval between the 25-th and 75-th percentile is shaded in turquoise in **b**, validating that this criterion satisfies the need that the values $\Delta\phi_t$ are close to zero. The number of random coincidences obtained by 50000 randomisations of the timings of the detected transitions using the probability density series and the mean time series are shown as histograms in **c**. At a statistical confidence of 5%, the threshold number of coincidences for significance is $N_{sig} = 191$ for the probability density series approach and $N_{sig} = 582$ for the mean time series approach. The corresponding observed number of coincidences N_{obs} equals 216 (581) for the probability density series (mean time series) approaches indicate that the probability density series approach offers a more robust and meaningful transition detection that allows us to validate the coincidence of hte transitions with phase-locking periods of the PDO and the ENSO.

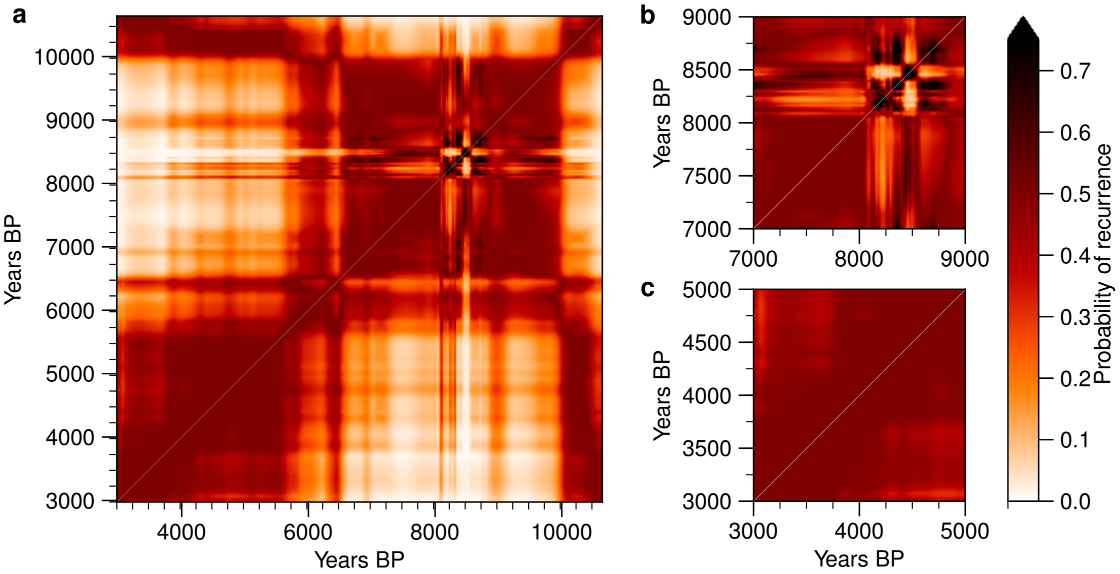

Supplementary Figure 9: Probabilty of recurrence matrix for Qunf cave, for the entire time series in **(a)**, for the '8.2k event' (for 7000–9000 yrs BP) in **(b)**, and for the '4.2k event' (for 3000–5000 yrs BP) in **(c)**. In contrast to the modular structure of the recurrence matrix in **(b)**, the recurrence plot in **(c)** is almost structureless, as all pairs of time points in this period have high probabilities of recurrence. The ubiquity of the high probabilities of recurrence in **(c)** can be traced back to the large uncertainties in the proxy record in the corresponding period during 3000–5000 yrs BP (see Supplementary Fig. 3c).

Supplementary Note 1 Correlation between two observables

Consider the classic Pearson's cross-correlation coefficient $Cor(X,Y)$ between two normalized observables X and Y (i.e., with mean zero and standard deviation one). In the typical time series framework, this is the averaged pairwise product of the measurements x_t and y_t,

$$Cor(X,Y) = \frac{1}{n}\sum_{t=1}^{n} x_t y_t. \qquad \text{(Supplementary Equation 1)}$$

In our proposed framework based on the density series $\{\varrho_t^X\}_{t=1}^N$ and $\{\varrho_t^Y\}_{t=1}^N$, we first note that the correlation of normalized variables is the expectation value,

$$Cor(X,Y) = \mathbf{E}[X,Y] = \iint xy\, \varrho^{XY}(x,y)\, \mathrm{d}x\, \mathrm{d}y, \qquad \text{(Supplementary Equation 2)}$$

where the joint density $\varrho^{XY}(x,y)$ is expressed in terms of marginals $\varrho^{XY}(x,y|t) := \varrho_t^{XY}(x,y)$ as $\varrho^{XY}(x,y) = (\sum_0^n \varrho_t^{XY}(x,y)\, \mathrm{d}t)/n$. If we now assume X and Y to be independent when conditioned on t, i.e., $\int \varrho_t^{XY}(x,y)\, \mathrm{d}t = \int \varrho_t^X(x)\varrho_t^Y(y)\, \mathrm{d}t$, the correlation coefficient is given by

$$Cor(X,Y) = \frac{\sum_{t=1}^{n} \bar{x}_t \bar{y}_t}{n}, \qquad \text{(Supplementary Equation 3)}$$

where $\bar{\cdot}_t$ denotes the expected value of the observable at time $T = t$ as obtained from $\varrho_t(\cdot)$. Pearson's correlation is thus properly estimable only if we know the joint density $\varrho_t^{XY}(x,y)$ or if X and Y are conditionally independent given T.

Supplementary Note 2 Probability density time series $\{\varrho_t^X\}_{t=1}^N$

The probability density series for the three real-world examples dealt with in our study are shown in Supplementary Figs. 1–3. In each case, the uncertainties arise from different sources. In the case of the financial stock indices (Supplementary Fig. 1), the uncertainties arise due to intra-day variations which fall below the daily sampling frequency of the dataset, and are hence approximated using a uniform random distribution of the stock index values between the reported daily minimum and maximum values. Even though the uncertainties appear to be negligibly small when compared to the drift in the index values over the span of the entire dataset, we note that intra-day fluctuations can be sufficiently high at times of financial crashes and volatile periods. This is an important feature that is missed in typical point-like time series representations. For the Niño 3.4 SST anomalies (Supplementary Fig. 2), the uncertainties arise due to the spatial heterogeneity of the SST values over the geographical grid demarcating the Niño 3.4 region in the equatorial Pacific. The distributions are thus estimated by pooling the monthly averaged SST values from all grid points for a given month into one sample and estimating their distribution using a kernel density estimate. In the case of the paleoclimate datasets, the uncertainties arise out of the imprecision in determining the age of the climate proxies. In most cases, it is important to note that the estimated distributions (Supplementary Fig. 3) are rather non-Gaussian, often skewed, and exhibit non-negligible variance.

Supplementary Note 3 Transitions detected using mean time series

The primary focus of our study is to emphasise the advantages of considering the probability density time series in lieu of a typical point-like time series. As done for the synthetic example (Fig. 2 of main text), we compare the results of the detection of abrupt transitions from our proposed approach on the density series to transitions detected when we use the mean value time series instead (Supplementary Figs. 4–6). In the case of the financial datasets (Supplementary Fig. 4), we see that the numbers of detected transitions are much higher when we use the mean time series, which we interpret as a lack of specificity, noting in particular that for the BSE SENSEX data, the entire period from mid-2007 to mid-2012 is filled with numerous 'transitions'. Even in the Niño 3.4 example (Supplementary Fig. 5.), employing the mean time series to the transition detection method results in an almost contiguous series of 'transitions' throughout the entire timespan of the data. However, when we look at the results for the Dongge cave in the paleoclimate example (Supplementary Fig. 6), we see that the results from the mean time series fail to detect the previously reported events at 4200 yrs BP and 8200 yrs BP (the famous '4.2k' and '8.2k' events). The impact of considering the mean time series on the final results of the abrupt transition detection may vary on the type, nature and magnitude of the uncertainties of the datasetes: While in some cases, (possibly random) fluctuations are detected as transitions, in others, large uncertainties (or a change in the underlying distribution) may wash out the transition signal in the expectation value. This further underscores the necessity for the proper representation and analysis of uncertainties in time series. Without proper care of the uncertainties, significant events might be missed out, but also non-significant events might be overemphasised.

Supplementary Note 4 Impact of uncertainties on transition detection

In the main text, we use the example of paleoclimate datasets to illustrate how uncertainties, when properly incorporated into the analysis, can impact the results of an abrupt transition detection scheme. We note how, in the case of the 4.2k event, we detect an abrupt transition around this time in the Dongge adn Tianmen speleothem records, but not in the Qunf cave record. The reason for not detecting this abrupt transition in the Qunf cave becomes clear from the estimated adjacency matrix $\hat{\mathbf{A}}$ network of recurrence probabilities (Supplementary Fig. 9a). In Supplementary Fig. 9b and Supplementary Fig. 9c, we magnify the probabitliies of recurrence around the 8.2k event and around the 4.2k event respectively. All pairs of time points around 4200 yrs BP have almost uniformly large probabilities of recurrence, such that there is not clear community (modular) strucure in the portion of the adjacency matrix represented in Supplementary Figure 9c. Contrast this to the situation around 8200 yrs BP, shown in Supplementary Fig. 9b, where a clearer community structure is visibile which increases the statistical confidence with which we can infer abrupt transitions in this period. This is not possible in the time period around 4200 yrs BP. Furthermore, the primary reason for the uniformly large probabilities of recurrence around 4200 yrs BP is that the Qunf cave time series has large uncertainties in the period around 3000–5000 yrs BP (Supplementary Fig. 3c). During this period, when we choose a pair of time points and ask the question: 'Did the observable recur at these time points?' we almost always come up with a non-negilible probability that they might have recurred. To put it

in other words, it takes higher precision (i.e., less uncertainty) to say confidently that two time points have not recurred. As a result, we get almost equally high probabilities of recurrence for all pairs of time points in the priod ca. 3000–5000 yrs BP for the Qunf cave time series.

This example illustrates how uncertainties can influence the transition detection results, and consequently influence all downstream analyses or inferences which are based on the list of transitions obtained. Proper representation of time series uncertainties and a thorough analysis which keeps the uncertainties in mind at each step along the way is thus an absolute necessity to avoid pitfalls in understanding abrupt transitions and their spatio-temporal characteristics.
